# Social networks of health care providers and patients in cardiovascular risk management: a study protocol

**DOI:** 10.1186/1472-6963-14-265

**Published:** 2014-06-18

**Authors:** Naomi Heijmans, Jan van Lieshout, Michel Wensing

**Affiliations:** 1 Radboud University Medical Centre, Nijmegen, The Netherlands; 2 Scientific Institute for Quality of Healthcare, PO 9101, 6500 HB Nijmegen, The Netherlands

**Keywords:** Social network analysis, Information sharing, Primary care, Cardiovascular disease

## Abstract

**Background:**

In recent years, preventive and clinical interventions for cardiovascular risk management have been implemented widely in primary care in the Netherlands. Although this has enhanced quality and outcomes of cardiovascular risk management, further improvement remains possible. In the planned observational study, we aim to examine the role of social networks of healthcare providers and patients in quality and outcomes of cardiovascular risk management.

**Methods/Design:**

In a longitudinal observational study, data on social networks of approximately 300 primary care providers from 30 general practices and 900 cardiovascular patients will be collected twice, with a six month interval, using a mix of measures. Social networks are documented with specifically designed questionnaires for patients, relatives, and healthcare professionals. For each included patient, we will extract from medical records to gather data on clinical processes and cardiovascular risk predictors. Data on self-management and psychosocial outcomes of patients will be collected using questionnaires for patients. The analysis focuses on identifying network characteristics, which are associated with (changes in) cardiovascular risk management or self-management.

**Discussion:**

This research will provide insight into the role of social networks of patients and providers in cardiovascular risk management in primary practice.

**Trial registration:**

Nederlands Trial Register NTR4069.

## Background

Cardiovascular disease (CVD) remains an important cause of mortality and reduced quality of life worldwide [1]. In 2010, CVD was the number one cause of death among women and the second cause of death for men in the Netherlands [2]. A wide range of preventive and clinical interventions targeting the population and patients at risk are recommended in prevailing practice guidelines, emphasizing comprehensive cardiovascular risk management (CVRM), life style changes, and preventive drug therapy [3]. In recent years, many conditions for providing recommended CVRM have been optimized in the Netherlands. These include the publication of a multidisciplinary clinical guideline and organizational standards for general practices, introduction of nurses in general practices, nationwide supply of paper-based and online patient education tools for CVD-patients as well as the general public, and targeted reimbursement for chronic illness care in primary care [4]. While these developments have improved CVRM, a specific number of patients still did not completely receive recommended CVRM or did not reach target values of CVRM [5]. So, the question is what additional determinants of CVRM and outcomes can be identified and addressed to optimize cardiovascular risk management.

Social network analysis (SNA) offers a new perspective on the implementation of evidence-based practice in healthcare. Previous research, which used SNA in health care, showed that social networks of patients and healthcare professionals could be measured in a valid way and showed substantial variation [6-8]. Pilot studies in primary care in the Netherlands have confirmed the feasibility and viability of specific measures for documenting social networks of information sharing [9-11]. Despite these and other studies in health care, insights into network-related mechanisms underlying healthcare delivery and self-management (health-related behaviors) are still limited. In this study, we will explore the role of social networks of healthcare providers and patients in the delivery and outcomes of CVRM in primary care. Social networks will be constructed using data on information exchange between individuals involved in CVRM.

### Theoretical background

The potential relevance of social networks for health outcomes is illustrated by a number of studies, which suggested that health-related behaviors are not randomly spread in a population but associated with social network structures [12-14]. This has led to the notion of ‘contagion processes’ in social networks, which seem to apply to a range of items, including infections, information, and behaviors. The underlying mechanisms of contagion patterns are heterogeneous, depending on the item of interest. For spread of information and behaviours, psychological mechanisms such as imitation of successful behaviours, role modelling, social comparison and selection of contacts can be drivers of contagion.

Contagion processes can be either simple or complex. In simple contagion processes, a single encounter has direct consequences. If applied to the transfer of information, the assumption is that access to information is the main enabler of uptake. Given the wide range of available information sources in health care, including many on the world wide web, and resulting information overload for both health professionals and patients, simple contagion may currently be rare. In many cases, information needs to be selected, prioritized and positively labeled to be put into action. Thus, a single exposure is often not sufficient to lead to change of knowledge or behaviors. Complex contagion represents spread of items under the influences of intensive, repeated and valued contacts. The assumptions of complex contagion are consistent with a number of theories. For instance, the Diffusion of Innovation Theory proposes that uptake of information occurs in most individuals by informal, personal contacts rather than through formal education [15].

**Figure 1:**
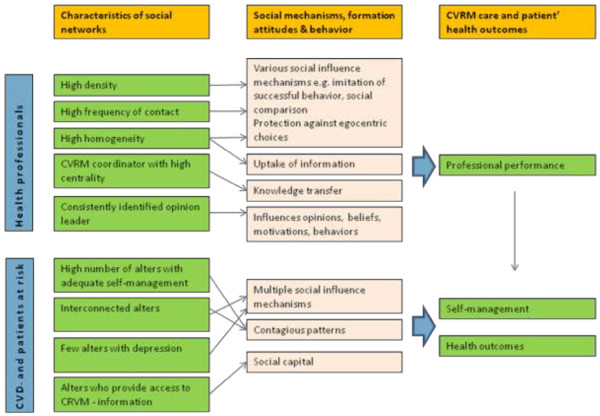
Hypothesized relations.

Drawing on previous research and theories on social networks and translation of information contagion into attitudes and behaviors, several network characteristics which may influence the delivery of primary care for CVRM by health professionals and self management of patients at risk for CVD and with established CVD can be inferred. For a schematic representation of the hypothesized relations see Figure 1. For healthcare professionals we expect a number of social network characteristics to be of importance. Firstly, a high density (the proportion of all possible connections in a given network that are actually present) is expected to be beneficial as it provides multiple opportunities for various social influence mechanisms, such as for example imitation of successful behaviors and social comparison [8,16,17]. Secondly, a high frequency of contacts (within existing relationships) is expected to be important, as this enhances social influence and offers protection against egocentric choices [16,18,19]. Thirdly, high homogeneity of individuals in a network (e.g. regarding educational background) is expected to enhance social comparison uptake of information items that are disseminated in the network [20]. Fourthly, individuals with high centrality in a social network are expected to be influential as they have many connections and high centrality has been associated with enhanced knowledge transfer [21,22]. In healthcare, individuals with high centrality are expected to be present in social networks as CVRM-coordinators or case managers. Both are purposefully created to become highly central individuals in healthcare delivery networks. Fifth, an informal opinion leader may be identifiable in a network. He or she represents a person who influences opinions, attitudes, beliefs, motivations, and behaviors of others [23]. For these network-related factors, it may be noted that the direction of the social influence (e.g. better or worse CVRM) depends on the content of the information that is shared.

For patients we also expect network characteristics to be important for and related to self-management (that is, health behaviors for diet, physical activity, and smoking). Firstly, the presence of a high number of individuals with favorable self-management as previous research showed that self-management behaviors (e.g. smoking, alcohol use, obesity) are clustered within networks [12-14]. Secondly, the effect of having individuals with appropriate self-management within ones network should be more profound when these individuals are also connected to each other, with resulting opportunities for multiple social influence mechanisms [24]. Thirdly, networks which contain few individuals with depressive symptoms may be positively related to self-management, as depression has shown to have both a contagious pattern in networks [25] and to be related to worse health behaviors [26,27] and impaired efforts for achieving lifestyle change [20]. Fourthly, the presence of persons who can provide information on CVRM in patients’ informal networks (e.g. nurses or physicians) adds to the social capital of an individual and can be expected to enhance self-management [28].

### Research aims

The overall aims of the study are:

1. To describe social networks of health care professionals in primary care involved in CVRM as well as those of patients with established CVD or a high risk thereof using a social networks approach.

2. To explore which characteristics of social networks are linked to key aspects of the delivery of high-quality CVRM (that is, providing advice and recommended treatment) and of patients’ self-management.

3. To explore the influence of several network characteristics on changes in delivery of high-quality CVRM and in patient’s self-management.

4. To evaluate different methods for including patients in a study of social networks.

### Key hypotheses

In summary, the following key hypotheses will be explored in the planned research project:

#### Hypotheses on health professionals

Patients are more likely to receive recommended CVRM and reach CVRM targets in general practices which have social networks characterized by: high density, high frequency of contact, high homogeneity, an appointed CVRM-coordinator who has a high degree of centrality, and a consistently identified opinion leader for CVRM.

#### Hypotheses on patients

Patients are more likely to have favorable self-management and reach CVRM targets if they have social networks which contain: a high number of individuals with adequate self-management, a high number of individuals with adequate self-management who are connected to each other, few individuals with depressive symptoms, and individuals who provide access to individuals who can provide information on CVRM, particularly health professionals (such as nurses or physicians).

## Methods

### Study design

This observational research is part of the ‘Tailored Implementation for Chronic Diseases’ (TICD) project. The TICD-project has the overall aim to develop and test methods of tailoring implementation interventions to determinants of practice in chronic illness care in five different chronic conditions in five different countries in Europe [29].

This study will be performed parallel to a two-arm RCT as developed by the Dutch team (trial registration: NTR4069). This approach is chosen as it will allow for both the investigation of network characteristics for quality of current CVRM care by health professionals and self-management of patients and for associations of network characteristics with changes in quality of CVRM care and self-management after completion of the RCT intervention program. The RCT aims at enhancing primary care for CVRM by improving professional performance of practice nurses [30]. Practice nurses conduct care for patients with chronic conditions. They provide patients with education and guidance about their disease, medication-use, and changes in lifestyle and independently perform consultation hours and regular checkups.

For this RCT, comparing an intervention vs a control (postponed intervention) group, a tailored intervention package has been developed which offers practice nurses several options for enhancing their knowledge on CVRM and counseling skills. Additionally, the package provides recommendations for referral of patients. This package consists of training and feedback on motivational interviewing technique and an e-learning program on CVRM. Also, practice nurses will be advised to pay particular attention to the presence of depressive symptoms and plan action according to these as patients with CVD have a higher risk for experiencing depressive symptoms [27] with concomitant impairments in altering self-management [20]. More specifically, practice nurses are recommended to refer patients without depressive symptoms to e-health learning modules, patients with mild depressive symptoms to a physical exercise group, and patients with severe depressive symptoms to psychological help as appropriate in their practice [30]. Data on professional performance of practice nurses, patients’ self-management and treatment outcomes will be measured at baseline and follow-up at six months after the start of this intervention [30].

In the present research we will measure characteristics of social networks of health professionals and patients at the start of the intervention program and after completion of it. Social networks will be constructed on information exchange on CVRM purposes. Data on professional performance of health professionals and self management and CVRM treatment targets of patients will be collected by patients’ medical file extraction, self-report questionnaires and telephone interviews. Additionally, we will measure network characteristics of so called ‘alters’ of health professionals and patients. An alter represents an individual who has contact with the person under investigation, typically family members for patients and other healthcare providers for healthcare professionals.

### Ethical approval

The Medical Ethical Committee of Radboud University Nijmegen Medical Centre has waived approval for both the social network study and the RCT study [30].

### Sample

The sample will consist of health care professionals, patients with high risk for CVD or established CVD and their alters.

#### ***Health care professionals***

We will include all health care professionals working in general practices participating in the RCT and who are involved in patient care (typically, general practitioners, practice nurses, and practice assistants). Based on power calculations in the RCT, 30 practices are planned to be included.

#### ***Patients***

Eligible patients are adults aged 18 or older, with high risk of CVD or established CVD and who are capable of providing informed consent [30]. Patients with high risk for CVD have a risk score of 20% or higher of 10-years-morbidity and mortality due to CVD. International Classification of Primary care (ICPC) codes will be used to extract eligible patients from medical records. Exclusion criteria are: diabetes mellitus, pregnancy and lactation, terminal illness, cognitive impairments, and poor language skills. Per practice we plan to include 15 patients with established CVD and 15 at high risk.

#### ***Alters***

*Alters of health care professionals* will comprise individuals with whom information on CVRM is exchanged but who are not part of the general practices’ network, as well as opinion leaders from outside general practices. We aim to include all alters of health care professionals.

*Alters of patients* include key individuals for information exchange, which most likely will be spouses or children of patients. We aim to include up to four persons considered to be important for dealing with patients’ condition or disease as indicated by the included patients in our measurements.

### Recruitment process and data collection

**Figure 2:**
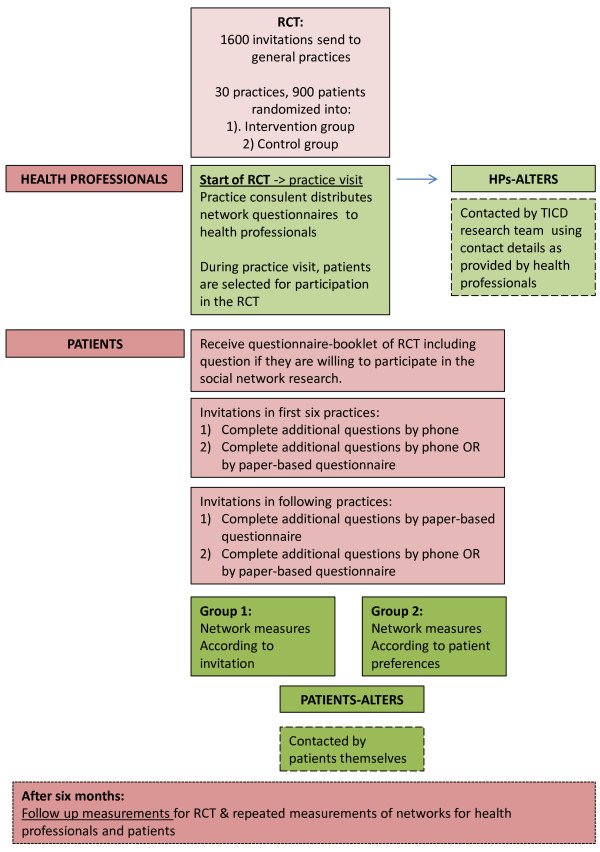
Study flow chart.

Specific details regarding the inclusion procedure of general practices and patients in the intervention are described in the protocol of the RCT [30]. For the RCT, all interventions and data collection procedures were planned to be performed from July 2013 until June 2014. The recruitment process and data collection procedures on behalf of these network studies will be performed parallel to the data collection of the RCT. Data collection procedures for social networks of health professionals and patients were planned in the same period, with some extension for patients needed until July 2014 because of our invitation procedures (see p8 for details). As data collection procedures for alters of health professionals start after receipt of completed questionnaires of health professionals and patients themselves, we expect to complete these procedures in August 2014. For a flow chart of the studies, see Figure 2.

#### ***Health care professionals***

The RCT will start with an outreach visit in which a comprehensive explanation of the RCT study and network measurements on behalf of the present research will be provided to practice nurses. Outreach visits will be performed by a trained practice consultant. A practice consultant is an expert in coaching and advising general practices in processes of change and improvement. Before these practice visits, names of persons who are involved in patient care in general practices will be derived online and checked for accuracy by practice nurses and will be used to generate personalized network rosters. These network questionnaires will be handed over to practice nurses during practice visits so that she or he can spread these to other health professionals within the general practice. Health professionals within general practices typically will be general practitioners, practice nurses, and practice assistants. A letter which contains comprehensive information on the network study will be enclosed to the questionnaire. Prepaid envelopes will be provided for returning filled out questionnaires.

#### ***Alters of healthcare professionals***

For including alters of health professionals, names, profession, and contact details will be extracted from completed network questionnaires of health professionals working within general practices. Invitation letters and network questionnaires will be mailed to alters within two weeks after receiving these completed questionnaires. When none or insufficient contact details are provided, we aim to collect these by using an Internet-search. Criteria entered in the Internet search will vary depending on information provided. We mainly will use Google for finding details needed.

#### ***Patients***

Patients will be selected from the general practices’ electronic data base using ICPC codes during practice visits of the RCT. They will receive a questionnaire booklet at the start and end of the intervention period on behalf of the RCT [30]. For inclusion in the network study, we will include three basic network questions (see Additional file 1) in this questionnaire booklet followed by an invitation for participation in the network study. An informed consent form will be enclosed and will be returned along with the questionnaire booklet of the RCT. During the inclusion of the first six practices, patients will be randomized into one of two groups. The first group will receive an invitation in the questionnaire booklet to participate in the network study by a telephone interview. The second group will be invited by the choice to participate by 1) a telephone interview *or* 2) a paper-based questionnaire. After inclusion of these practices we will invert the design for another six practices so that the first group will receive an invitation to participate in the network study by a paper-based questionnaire and the second group will be invited by providing the choice to participate by either 1) a paper-based questionnaire or 2) a telephone interview. These response groups are included so that we will be able to compare which approach is most feasible for including patients in network measurements as current research on this is scarce. For inclusion of patients from additional practices, we will employ the inclusion method as assessed to be most feasible. The basic questions are incorporated in the RCT questionnaire to ensure that basic data for all patients is included. Questionnaires will be send and interviews will be held approximately two weeks after receipt of accepted invitations.

#### ***Alters of patients***

Patients are asked to spread questionnaires to their alters, for which specific instructions will be provided in their own questionnaires or during interviews. For this purpose, patients will receive four additional questionnaires; one for their personal opinion leader (specified on network questionnaires as ‘the person considered to be most important for dealing with condition or disease’), and three for ‘important others’ (specified as ‘other persons considered to be important for handling condition or disease’) with accompanying information letters and informed consent forms. We have chosen to include four alter-questionnaires as limited current research indicates that this is the maximum number of important or significant others within patients’ networks [31]. Patients and alters can contact TICD researchers for additional questionnaires if desired.

##### Reminder procedures

In case of no response from healthcare professionals, we will ask practice nurses to remind non responding persons in their general practice. In case of no response from patients we will send letters as reminders.

### Outcomes & measures

#### ***Primary outcomes for health professionals***

Baseline and 6-month follow-up measures will be conducted for health care professionals, patients, and their alters.

### Health care professionals and their alters

Main outcomes for health professionals will consist of 1) the description of social networks and 2) the hypothesized effects of several social network characteristics, controlled for possibly confounding variables, on professional performance of practice nurses and patients’ health outcomes. Additionally, exploration of effects on social network characteristics on changes in professional performance after completion of the RCT are of main interests.

#### 

**Professional performance** Professional performance reflects application of recommendations for personalized counseling and education of CVRM patients by practice nurses. Professional performance is represented with a dichotomous score, reflecting adequate or inadequate performance. We will consider personalized lifestyle advice as provided by practice nurses to be adequate when at least one of the following conditions is met:

1. There is a record in the patients’ medical file or other healthcare provider-based records that the patient has received advice on at least one lifestyle item as specified in prevailing guidelines of CVRM; diet, smoking or physical exercise, and which has been relevant for the individual patient in the previous six months. At least one target, made up maximally 15 months ago, for improving an aspect of lifestyle should be recorded. Also, practice nurses are required to make a register note when a patient has an adequate lifestyle.

2. There is a notation in the patients’ medical file that the patient has none, mild or major depressive symptoms and that the patient has been referred to E-health, a physical exercise group, or depression treatment respectively.

#### ***Other measurements on health care professionals will include***

##### 

**Descriptive variables** Descriptive measures will include size of practice (number of staff and patients), profession, and involvement in other organizations or projects and will be measured using the Epa Cardio abstraction tool for medical audit instrument [32].

##### 

**Information items for constructing social networks** For constructing social networks we will use a specifically developed and personalized roster questionnaire (see Additional file 2: Appendix 2a for the network questionnaire for health professionals from general practices participating in the RCT and Additional file 2: Appendix 2b for the network questionnaire of alters of health professionals). On this network roster, health professionals will be asked to indicate their social contacts for CVRM information sharing and receiving on two subjects:

1) medical policy for CVRM in general and on 2) CVRM for specific patients. In this network roster, names are listed of all persons involved in patient care within a general practice so that respondents can tick names of persons with whom they share information. Also, space is provided for names of persons outside the general practice, respondents are asked to fill out these names themselves.

##### 

**Frequency of contact** For measuring frequency of contact for information sharing, health professionals are asked to indicate whether they have been in contact on a 1) daily/weekly or 2) monthly/yearly basis with each person they share information with.

##### 

**CVRM-coordinators** For the identification of the presence of CVRM-coordinators within general practices we will ask health professionals to list the name(s) of the person(s) responsible for coordination of CVRM within the particular general practice.

##### 

**Opinion leaders** For the identification of opinion leaders we will ask health professionals to write down the name and occupation of the person they consider to have ‘a significant influence on their current practice in CVRM’. Health professionals will be instructed that this person can be anyone from inside or outside the general practice, and that the influence this person has had can be either current or from the past.

##### 

**Priority for preventive treatment and CVRM targets** For determining whether social network characteristics are related to certain attitudes on clinical processes for CVRM, and whether these attitudes are more or less present in certain social networks, we will measure priority for preventive treatment and achievement of CVRM targets by presenting five statements containing general recommendations and targets for CVRM (e.g. ‘for patients with a high risk for CVD, strive for a systolic blood pressure < 140 mmHg’). These items were selected from current guidelines and previous research indicated [32] that primary care for these items can be enhanced. Health professionals are asked to indicate on a 5-point Likert scale (1: totally unimportant – 5: highly important) how important they consider ‘a change in direction of the presented target’ as indicator for priority for preventive treatment and ‘achievement of the presented target’ as indicator for priority for CVRM targets.

### Primary outcomes for patients

#### ***Patients and their alters***

Main outcomes for patients will consist of 1) description of social networks, 2) response rates after different invitations for participation in the network study, and 3) the hypothesized effects of several social network characteristics, controlled for possibly confounding variables, on self-management and health outcomes. Additionally, exploration of effects of social network characteristics on changes in self-management and health outcomes after completion of the RCT are of main interest.

##### 

**Response rates** One of the main outcomes for patients are response rates of including patients by inviting them with either 1) an invitation with one option to participate in the social network study or 2) an invitation to participate in the research with two options so that patients can indicate their preference for participating by a telephone interview or a paper-based questionnaire.

##### 

**Self-management** Self-management will be measured using a composite questionnaire on physical activity; *Rapid Assessment of Physical Activity (RAPA), 9 items*[33], diet; *reduced Rapid Eating and Activity Assessment (REAP-s), 12 items*[34], and smoking; *(MID-SIZED Model), 8 items*[35].

Scores on the RAPA ≥ 5 indicate sufficient physical activity. The REAP-s asks how often persons engage in particular diet patterns in a regular week (e.g. high sugar/calorie sweets and beverages intake), using four response categories (‘often/usually’, ‘sometimes’, ‘rarely’, and (for items where appropriate) ‘not applicable’). Patients who score ≤ 5 items on the REAP-s as ‘often/usually’ will be considered to have a healthy diet. Current smoking status is indicated by a dichotomous score (yes/no).

##### 

**Health outcomes** Health outcomes will consist of SBP, cholesterol, and risk score. Thresholds for desired treatment values for SBP and cholesterol may differ for patients at risk, with established CVD, patients of certain ages, and according to individual targets set in agreement with treating health professionals and will be analyzed accordingly. Risk scores, only applicable to patients without established CVD, will be calculated using prevailing risk estimation tables [36]. The following parameters will be used in the calculation: age, gender, smoking status, systolic blood pressure (SBP) and total cholesterol/HDL-cholesterol ratio. The Epa Cardio abstraction tool [31] for medical audit will be used for abstracting these health outcomes.

#### ***Other measures on patients will include***

##### 

**Descriptive variables** Descriptive measures will include age, sex, ethnicity, marital status, educational level, and social economic status (SES). For data collection we will use a questionnaire containing items from the Epa cardio abstraction tool [32].

##### 

**Information items for constructing social networks** For constructing and measuring social networks, patients are asked to indicate on a network roster from which persons they have received information on 1) medical treatment, 2) handling their condition or disease, and 3) practical help. We will provide a specifically developed and personalized roster questionnaire with names and disciplines of health professionals from their general practices (see Additional file 3: Appendix 3a). Patients are offered the possibility to add names of other persons (healthcare professionals/people in personal circle) they have received information from.

Alters are asked to indicate on a network roster to which persons they have provided information on 1) medical treatment, 2) handling their condition or disease, and 3) practical help using a specifically developed and personalized roster questionnaire (see Additional file 3: Appendix 3b).

##### 

**Frequency of contact** For measuring frequency of contact, patients will be asked to indicate how often they have received information from persons who have provided information in the last year. Similarly, alters will be asked how often they have provided information.

##### 

**Central care providers** A central care provider represents the health professional patients will contact first when they have questions regarding a specific condition or disease and who coordinates care when multiple health professionals are involved in treatment.

For identification of central care providers we will ask patients to write down the name of the person they consider to be their central care provider for CVRM, specified as ‘the first person they would approach in case of uncertainties or troubles regarding their condition or disease’.

##### 

**Opinion leaders** For identification of opinion leaders patients will be asked to write down the name of the person they consider ‘to be most important for dealing with disease or lifestyle’. Patients will be instructed that this person doesn’t need to be ‘most important’ for a specific reason and that this person doesn’t need to be part of the patient’s personal environment.

##### 

**Number of alters with appropriate self-management** For assessing what number of alters in patients’ networks have adequate self-management we will ask patients to indicate whether he/she believes that their indicated alters smoke, have a healthy diet, and engage in sufficient physical activity. In addition, we will ask alters of patients to complete questionnaires on their lifestyle habits (smoking; *(MID-SIZED Model, 8 items*[35]), diet; *reduced Rapid Eating and Activity Assessment (REAP-s), 12 items*[34], and physical activity; *Rapid Assessment of Physical Activity (RAPA), 9 items*[33].

##### 

**Connectedness of alters** For assessing whether alters of patients are also connected to each other, we will use information from the network rosters completed by all alters of a given patient.

##### 

**Number of alters with depressive symptoms** For assessing whether alters with depressive symptoms are present in patients’ networks we will ask alters of patients to complete the Patient Health Questionnaire (PHQ-9) [37], which is a short questionnaire for depression.

##### 

**Number of alters providing access to CVRM information** For assessing how many persons who can provide access to CVRM information are available in patients’ social networks we will ask patients to indicate how many of the persons from whom they receive CVRM related information have a medical occupation or have been educated for this.

##### 

**Personal characteristics** For assessment of personal characteristics of patients we will measure patient activation (*Patient Activation Measure, PAM*[38]), therapy adherence (*Medication Adherence Measure*[39]), quality of life (*EQ-5D*[40]), and depressive symptoms (*Patient Health Questionnaire, PHQ-9*[37]). Alters of patients will complete the PHQ-9. Higher total scores on these measures will indicate higher patient activation, therapy adherence, quality of life, and depressive symptoms respectively.

### Sample size calculation

The RCT study, in which the present research is embedded, is powered to detect a 15% difference in provided personalized lifestyle advice by including 900 patients clustered within 30 practices [30]. For the present research, we calculated the sample size for detecting a 15% difference in response rate of patients who are approached by 1) an invitation for participation in this study with a telephone interview) and 2) the choice for participating by a telephone interview or a paper-based test. Assuming alpha is 0.05, power of 0.80, a response rate of 50% in the first group and 65% in the second group, we estimated that we need to include 338 patients.

### Data analysis

We will use UCINET for constructing and obtaining social network parameters of general practices on broad and specific information exchange and general information receipt networks of patients. The statistical package R will be used for all other analyses. All data analyses will be based on ‘intention to treat’.

### Construction of network characteristics

*Density* represents the proportion of all possible connections in a network that are actually present. *Homogeneity* represents the similarity of persons within clusters. Similarity of persons will be assessed regarding priority given to preventive treatment and CVRM targets, and other individual characteristics. *Centrality* is a measure for the extent to which a network is organized around a single person and can be divided into in- and outdegree centralization. The first specifies information flow from various network members to a single person, the latter whether information flows from a single person to the other network members.

### Statistical analyses

Response rates for health professionals and patients will be determined. For patients we will compare response rates between the patient group invited by either a single option for participation and the group that will be offered the choice to participate by either a telephone interview or a paper-based test in order to assess which method is most feasible for including them in social network research using *X*^2^ tests.

Reliability of reported social network connections will be investigated by examining the proportion of all possible connections that will be mutually reported present or absent (reciprocity coefficients in non-directed networks). In accordance with guidelines on handling missing values, we will substitute these by values as provided by responses of other individuals. In case of no information on connections, we will indicate no contact by a filling in a zero in the data [41].

For describing social networks of health professionals and patients we will compute network parameters and provide visualization by using graphic displays. Random permutation tests will be used for comparisons of network characteristics.

For testing our hypotheses and further explorative analyses, controlling for potential confounders, we will use multivariate logistic regression models to investigate the effect of social networks characteristics on professional performance of practice nurses and patients’ self-management and health outcomes.

In the analysis of professional performance of practice nurses regression models will include six network characteristics (density, frequency of contact, homogeneity, presence of informal opinion leaders and CVRM-coordinators, and centrality) as predictors of main interest. As control variables we will include trial arm (intervention vs control group), personal characteristics (amongst others education, years of experience in profession), and practice characteristics (practice size, involvement in other projects or organizations).

In the analyses of self-management and health outcomes in patients, several multivariate logistic regression models will be estimated. For self-management, separate models will be estimated on physical activity, diet, and smoking. For health outcomes, separate models will be estimated on SBP, cholesterol, and risk score. Models will include four network characteristics as predictors: number of alters with favorable health behaviors, who are also connected among each other, number of alters with depressive symptoms, and number of alters who can provide CVRM-related information. The regression models will be controlled for trial arm (intervention vs control group), patient group (high risk or established CVD), and several personal characteristics: age, sex, ethnicity, marital status, SES, patient activation, therapy adherence, and depressive symptoms.

Two approaches will be used to investigate effects of social network characteristics on change in professional performance of practice nurses and self-management and health outcomes of patients after the intervention period at six month follow-up. First, follow-up outcomes measures will be treated as dependent variables. We will estimate similar models as described above and include baseline measures of outcomes of interest as predictors. Second, we will consider change as the difference between the baseline- and follow-up measures of the diverse outcomes of interest. Ordinal logistic regression models will be estimated, with change represented in three categories; a shift from inadequate to adequate outcomes, no change, and shift from adequate to inadequate outcomes.

## Discussion

Although many efforts to improve primary care for CVRM have been conducted, recent research shows that improvements remain possible [32]. The wide range of approaches aimed at improvement in primary care signals the need for the identification of new opportunities for enhancing CVRM. Social network analysis may be a promising approach to provide these needed new insights. The results of this study can be of practical importance for clinicians and policymakers involved in maintaining and enhancing quality of health care by enhancing the understanding of certain characteristics of networks and their associations with positive or negative quality of CVRM care. Such insights can provide guidance to efforts aimed at improving functioning of networks. For example, if high homogeneity is found to be present in social networks and can be associated with high quality CVRM-care or change into this direction, future efforts aimed at enhancing health care can consider this network characteristic when composing health-care teams. Also, the results of this research can have a clinical importance by clarifying the role and importance of patients’ social environments for handling disease and maintaining or altering self-management. These insights can provide input for future research and interventions aiming at improving self-management of patients.

## Abbreviations

CVD: Cardiovascular disease; CVRM: Cardiovascular risk management; SNA: Social networks analysis; RCT: Randomized controlled trial; TICD: Tailored implementation for chronic diseases; SBP: Systolic blood pressure.

## Competing interests

The authors declare that they have no competing interests.

## Authors’ contributions

All authors contributed to the study design. NH wrote the draft version of this protocol which was commented on by JvL and MW. MW is the project leader of the TICD project. All authors critically assessed and approved the manuscript.

## Pre-publication history

The pre-publication history for this paper can be accessed here:

http://www.biomedcentral.com/1472-6963/14/265/prepub

## Supplementary Material

Additional file 1 Basic network questions patients. Click here for file

Additional file 2 **Appendix 2a Network questionnaire for health professionals.** Appendix 2b Network questionnaire for alters of health professionals. Click here for file

Additional file 3 **Appendix 3a Network questionnaire for patients.** Appendix 3b Network roster for alters of patients. Click here for file

## References

[B1] World Health Organization Noncommunicable Disease and Mental Health Cluster.: Integrated Management of Cardiovascular Risk: [report of a WHO meeting, Geneva, 9–12 July 2002] 2002 Geneva: WHO

[B2] Vaartjes I Nederlandse Hartstichting Hart- en vaatziekten in Nederland 2010: Cijfers over leefstijl- en risicofactoren, ziekte en sterfte 2010 Den Haag: Nederlandse Hartstichting

[B3] Fifth Joint Task Force of the European Society of Cardiology, European Association of Echocardiography, European Association of Percutaneous Cardiovascular Interventions, European Heart Rhythm Association, Heart Failure Association, European Association for Cardiovascular Prevention & Rehabilitation, European Atherosclerosis Society, International Society of Behavioural Medicine, European Stroke Organisation, European Society of Hypertension, European Association for the Study of Diabetes, European Society of General Practice/Family Medicine, International Diabetes Federation Europe, European Heart Network European Guidelines on cardiovascular disease prevention in clinical practice (version 2012): the Fifth Joint Task Force of the European Society of Cardiology and Other Societies on Cardiovascular Disease Prevention in Clinical Practice (constituted by representatives of nine societies and by invited experts) Eur J Prev Cardiol 2012 14 4 585 667 10.1177/2047487312450228 22763626

[B4] Van Lieshout J Wensing M Campbell SM Grol R Primary care strength linked to prevention programs for cardiovascular disease Am J Manag Care 2009 14 4 255 262 19355798

[B5] Ludt S Petek D Laux G Van Lieshout J Campbell SM Kunzi B Glehr M Wensing M Recording of risk-factors and lifestyle counselling in patients at high risk for cardiovascular diseases in European primary care Eur J Prev Cardiol 2012 14 2 258 266 21450582 10.1177/1741826711400510

[B6] Christakis NA Fowler JH Social contagion theory: examining dynamic social networks and human behavior Stat Med 2013 14 4 556 577 22711416 10.1002/sim.5408 PMC3830455

[B7] Keating NL O’Malley AJ Murabito JM Smith KP Christakis NA Minimal social network effects evident in cancer screening behavior Cancer 2011 14 13 3045 3052 21264828 10.1002/cncr.25849 PMC3119780

[B8] West E Barron DN Dowsett J Newton JN Hierarchies and cliques in the social networks of health care professionals: implications for the design of dissemination strategies Soc Sci Med 1999 14 5 633 646 10080364 10.1016/s0277-9536(98)00361-x

[B9] Weenink JW Van Lieshout J Jung HP Wensing M Patient Care Teams in treatment of diabetes and chronic heart failure in primary care: an observational networks study Implement Sci 2011 14 66 21722399 10.1186/1748-5908-6-66 PMC3143081

[B10] Wensing M van der Eijk M Koetsenruijter J Bloem BR Munneke M Faber M Connectedness of healthcare professionals involved in the treatment of patients with Parkinson’s disease: a social networks study Implement Sci 2011 14 67 21722400 10.1186/1748-5908-6-67 PMC3150321

[B11] Wensing M Van Lieshout J Koetsenruiter J Reeves D Information exchange networks for chronic illness care in primary care practices: an observational study Implement Sci 2010 14 3 20205758 10.1186/1748-5908-5-3 PMC2822738

[B12] Christakis NA Fowler JH The spread of obesity in a large social network over 32 years N Engl J Med 2007 14 4 370 379 17652652 10.1056/NEJMsa066082

[B13] Christakis NA Fowler JH The collective dynamics of smoking in a large social network N Engl J Med 2008 14 21 2249 2258 18499567 10.1056/NEJMsa0706154 PMC2822344

[B14] Rosenquist JN Murabito J Fowler JH Christakis NA The spread of alcohol consumption behavior in a large social network Ann Intern Med 2010 14 7 426 433 W141 20368648 10.1059/0003-4819-152-7-201004060-00007 PMC3343772

[B15] Rogers EM Diffusion of Innovations 1983 New York: Free Press

[B16] Granovetter M The impact of social structure on economic outcomes J Econ Perspect 2005 14 1 33 50

[B17] Centola D The spread of behavior in an online social network experiment Science 2010 14 5996 1194 1197 20813952 10.1126/science.1185231

[B18] Campbell E Salathe M Complex social contagion makes networks more vulnerable to disease outbreaks Sci Rep 2013 14 1905 23712758 10.1038/srep01905 PMC3664906

[B19] Centola D Macy M Complex contagions and the weakness of long ties Am J Sociol 2007 14 3 702 734

[B20] Kohlmann S Kilbert MS Ziegler K Schulz KH Supportive care needs in patients with cardiovascular disorders Patient Educ Couns 2013 14 3 378 384 23391685 10.1016/j.pec.2013.01.002

[B21] Shi XL Adamic LA Strauss MJ Networks of strong ties Physica A 2007 14 1 33 47

[B22] Van Wijk R Jansen JJP Lyles MA Inter- and intra-organizational knowledge transfer: a meta-analytic review and assessment of its antecedents and consequences J Manage Stud 2008 14 4 830 853

[B23] Valente TW Pumpuang P Identifying opinion leaders to promote behavior change Health Educ Behav 2007 14 6 881 896 17602096 10.1177/1090198106297855

[B24] Seeman TE Social ties and health: the benefits of social integration Ann Epidemiol 1996 14 5 442 451 8915476 10.1016/s1047-2797(96)00095-6

[B25] Rosenquist JN Fowler JH Christakis NA Social network determinants of depression Mol Psychiatry 2011 14 3 273 281 20231839 10.1038/mp.2010.13 PMC3832791

[B26] Atlantis E Shi Z Penninx BJ Wittert GA Taylor A Almeida OP Chronic medical conditions mediate the association between depression and cardiovascular disease mortality Soc Psychiatry Psychiatr Epidemiol 2012 14 4 615 625 21384119 10.1007/s00127-011-0365-9

[B27] Mastrogiannis D Giamouzis G Dardiotis E Karayannis G Chroub-Papavaiou A Kremeti D Spiliopoulos K Georgoulias P Koutsias S Bonotis K Mantzorou M Skoularigis J Hadjigeorgiou GM Butler J Triposkiadis F Depression in patients with cardiovascular disease Cardiol Res Pract 2012 14 794762 22830072 10.1155/2012/794762 PMC3398584

[B28] Song LJ Chang TY Do resources of network members help in help seeking? Social capital and health information search Soc Networks 2012 14 4 658 669

[B29] Wensing M Oxman A Baker R Godycki-Cwirko M Flottorp S Szecsenyi J Grimshaw J Eccles M Tailored Implementation For Chronic Diseases (TICD): a project protocol Implement Sci 2011 14 103 21899753 10.1186/1748-5908-6-103 PMC3179734

[B30] Huntink E Heijmans N Wensing M Van Lieshout J Effectiveness of a tailored intervention to improve cardiovascular risk management in primary care: study protocol for a randomised controlled trial Trials 2013 14 433 24341368 10.1186/1745-6215-14-433 PMC3895794

[B31] Rantanen A Kaunonen M Astedt-Kurki P Tarkka MT Coronary artery bypass grafting: social support for patients and their significant others J Clin Nurs 2003 14 158 166 10.1046/j.1365-2702.2003.00847.x 14723667

[B32] Campbell SM Ludt S Van Lieshout J Boffin N Wensing M Petek D Grol R Roland MO Quality indicators for the prevention and management of cardiovascular disease in primary care in nine European countries Eur J Cardiovasc Prev Rehabil 2008 14 5 509 515 18695594 10.1097/HJR.0b013e328302f44d

[B33] Topolski TD LoGerfo J Patrick DL Williams B Walwick J Patrick MB The Rapid Assessment of Physical Activity (RAPA) among older adults Prev Chronic Dis 2006 14 4 A118 16978493 PMC1779282

[B34] Segal-Isaacson CJ Wylie-Rosett J Gans KM Validation of a short dietary assessment questionnaire: the Rapid Eating and Activity Assessment for Participants short version (REAP-S) Diabetes Educ 2004 14 5 774 776 778 passim 15510530 10.1177/014572170403000512

[B35] Behavior change consortium, mid-sized model smoking measure https://commonfund.nih.gov/behaviorchange/index

[B36] Nederlandse Huisartsen Genootschap Multidisciplinaire richtlijn Cardiovasculair risicomanagement 2011 Den Haag: Bohn Stafleu van Loghum

[B37] Kroenke K Spitzer RL Williams JB The PHQ-9: Validity of a brief depression severity measure J Gen Intern Med 2001 14 9 606 613 11556941 10.1046/j.1525-1497.2001.016009606.x PMC1495268

[B38] Hibbard JH Mahoney ER Stockard J Tusler M Development and testing of a short form of the patient activation measure Health Serv Res 2005 14 1 1918 1930 16336556 10.1111/j.1475-6773.2005.00438.x PMC1361231

[B39] Morisky DE Green LW Levine DM Concurrent and predictive validity of a self-reported measure of medication adherence Med Care 1986 14 1 67 74 3945130 10.1097/00005650-198601000-00007

[B40] Brooks R Charro F Rabin R The measurement and valuation of health status using EQ-5D: a European perspective; evidence from the EuroQol BIOMED research programme 2003 Dordrecht [u.a.]: Kluwer

[B41] Kossinets G Effects of missing data in social networks Soc Networks 2006 14 3 247 268

