# Supplementary material for: Social networks of health care providers and patients in cardiovascular risk management: a study protocol
Source: BMC Health Serv Res. 2014 Jun 18;14:265. doi: 10.1186/1472-6963-14-265 (PMC4071149; doi:10.1186/1472-6963-14-265)
Supplement: Additional file 1 — Basic network questions patients. [file 1472-6963-14-265-S1.docx]

Please note that we use the term ‘condition’ in questions for patients with a high risk for CVD and ‘disease’ for patients with established CVD.

**Information regarding your condition or disease**

*We would like to ask you which persons gave you information regarding (a high risk for) cardiovascular disease.*

| **1.** | **Who gave you information about your condition or disease and its treatment?**  Please think about information of the treatment of your condition/disease, e.g. which medication you need, symptoms and risk factors of this condition/disease, and information about diet and physical activity. *Multiple answers are possible.* | |
| --- | --- | --- |
|  | General practitioner |  |
|  | Nurse or practice nurse from your general practice |  |
|  | Home care employee |  |
|  | Spouse |  |
|  | Son |  |
|  | Daughter |  |
|  | Friend |  |
|  | Neighbor |  |
|  | Acquaintance  Someone else, namely: …………………………………………… | |
|  |  | |

| **2.** | **Who gave you information about handling your condition or disease?**  Please think about information on e.g. handling concerns or problems you have because of your condition/disease, or about finding someone to talk about these. *Multiple answers are possible.* | |
| --- | --- | --- |
|  | General practitioner |  |
|  | Nurse or practice nurse from your general practice |  |
|  | Home care employee |  |
|  | Spouse |  |
|  | Son |  |
|  | Daughter |  |
|  | Friend |  |
|  | Neighbor |  |
|  | Acquaintance  Someone else, namely: …………………………………………… | |
|  |  | |

| **3.** | **Who gave you information about practical help for your condition or disease**  Please think about information regarding, for example, doing groceries or small tasks in and around your home, e.g. provided by volunteers, welfare workers, home care, or domestic help. *Multiple answers are possible.* | |
| --- | --- | --- |
|  | General practitioner |  |
|  | Nurse/practice nurse from your general practice |  |
|  | Home care employee |  |
|  | Spouse |  |
|  | Son |  |
|  | Daughter |  |
|  | Friend |  |
|  | Neighbor |  |
|  | Acquaintance  Someone else, namely: …………………………………………… | |
|  |  | |
