# Supplementary material for: Social networks of health care providers and patients in cardiovascular risk management: a study protocol
Source: BMC Health Serv Res. 2014 Jun 18;14:265. doi: 10.1186/1472-6963-14-265 (PMC4071149; doi:10.1186/1472-6963-14-265)
Supplement: Additional file 2 — Appendix 2a Network questionnaire for health professionals. Appendix 2b Network questionnaire for alters of health professionals. [file 1472-6963-14-265-S2.doc]

Appendix 2a


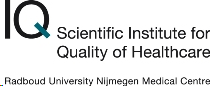

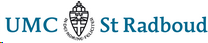


**Questionnaire for health professionals**


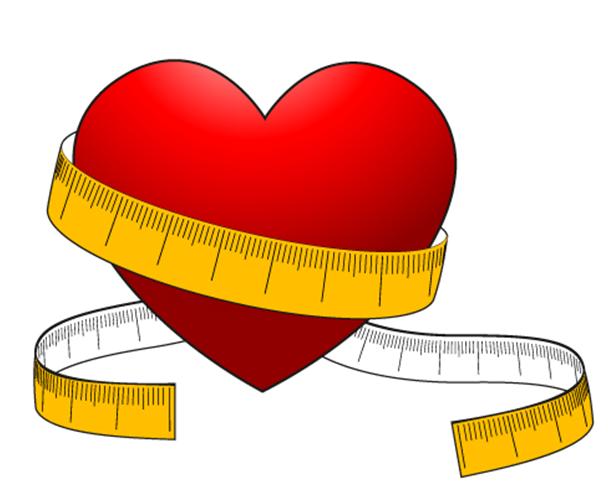


**Information networks**

**1.** We are interested in professional contacts between health care providers who are involved in cardiovascular risk management (CVRM). Please indicate in the scheme below to whom you have **given information** or from whom you have **receive information** in the past 12 months regarding:

1. Medical policy for CVRM in general, including special groups, such as patients of 80 years and older or with co-morbidity.
2. CVRM for a specific patient, e.g. when treatment is ineffective

- We also would like to ask you to indicate how often you’ve had contact for sharing information.

Please fill in a 1 for ‘daily or weekly’ and 2 for ‘monthly or yearly’

- We’ve already filled out names for persons from your general practice. Would you please fill out names for other persons you have shared information with?

|  | | **1.**  **Medical policy CVRM** | | How often contact for sharing information? | **2.**  **CVRM**  **For a specific patient** | | How often contact for sharing information? |
| --- | --- | --- | --- | --- | --- | --- | --- |
| **I gave information or received information from:** | | I gave to: | I received from: | 1= daily or weekly  2=monthly or yearly | I gave to: | I received from: | 1= daily or weekly  2=monthly or yearly |
| **Persons within your general practice:** | |  |  |  |  |  |  |
| GP1 | *Names* |  |  |  |  |  |  |
| GP2 |  |  |  |  |  |  |  |
| GP3 |  |  |  |  |  |  |  |
| PN1 |  |  |  |  |  |  |  |
| PN2 |  |  |  |  |  |  |  |
| PN3 |  |  |  |  |  |  |  |
| PA1 |  |  |  |  |  |  |  |
| PA2 |  |  |  |  |  |  |  |
| PA3 |  |  |  |  |  |  |  |
| **Persons outside your general practice:** | |  |  |  |  |  |  |
| Other GPs | *Names* |  |  |  |  |  |  |
| Other PNs |  |  |  |  |  |  |  |
| Other PAs |  |  |  |  |  |  |  |
| Dietician |  |  |  |  |  |  |  |
| Physical therapist |  |  |  |  |  |  |  |
| Psychologist |  |  |  |  |  |  |  |
| Cardiologist |  |  |  |  |  |  |  |
| Others, please specify: |  |  |  |  |  |  |  |
|  |  |  |  |  |  |  |  |

**1b.** In order to optimize knowledge on information flows of CVRM, we would to ask persons from outside your general practice some questions as well. Therefore, we ask you to provide contact details (address, telephone number, or an e-mail address) of these persons. We emphasize that this information will be treated confidentially.

If you prefer not to provide contact details, please skip this question.

Contact details:

|  |  |  |
| --- | --- | --- |
|  |  |  |
|  |  |  |

**2**. Who is responsible for coordination of CVRM in your practice?

|  |  |  |
| --- | --- | --- |
|  |  |  |
|  |  |  |

**2b**. We also would like to ask some questions to this person(s). Therefore, we ask you to provide contact details (address, telephone number, or an e-mail address) of these persons. We emphasize that this information will be treated confidentially.

If you prefer not to provide contact details, please skip this question.

Contact details:

|  |  |  |
| --- | --- | --- |
|  |  |  |
|  |  |  |

**3.** Can you name one person (not an organization) who you consider to have a significant influence on your current practice in CVRM? This person can be anyone from inside or outside your general practice, and the influence this person has had can be either current or from the past.

Please also provide a name an function.

| Name: |  |  |
| --- | --- | --- |
| Function: |  |  |
|  |  |  |

**3b**. We also would like to ask some questions to this person. Therefore, we ask you to provide contact details (address, telephone number, or an e-mail address) of this person. We emphasize that this information will be treated confidentially.

If you prefer not to provide contact details, please skip this question.

Contact details:

|  |  |  |
| --- | --- | --- |
|  |  |  |
|  |  |  |

**4.** We are interested in your opinion regarding recommendations for treatment of older patients (80 years and older) who receive care according to the CVRM guideline, and who have a limited life expectancy (less than 5 years).

Would you please indicate how important you consider:

- A change towards the treatment target, e.g. some reduction in blood pressure even though the treatment target has not been achieved
- Achieving the treatment target

Encircle a number: 1 = very unimportant, 2 = unimportant, 3 = neutral, 4 = important, 5 = very important.

|  | **Recommendation** | **Importance of change towards treatment target**  **(1-5)** | **Importance of achieving treatment target**  **(1-5)** |
| --- | --- | --- | --- |
| **1** | Aim at SBP < 140 mm Hg in patients with a 10 years risk of cardiovascular mortality or morbidity ≥ 20% | 1 2 3 4 5 | 1. 2 3 4 5 |
| **2** | Aim at SBP < 140 mm Hg for patients with CVD | 1 2 3 4 5 | 1. 2 3 4 5 |
| **3** | Aim at LDL cholesterol < 2.5 mmol/l in patients with a 10 years risk of cardiovascular mortality or morbidity ≥ 20% | 1 2 3 4 5 | 1. 2 3 4 5 |
| **4** | Aim at LDL cholesterol < 2.5 mmol/l for patients with CVD | 1 2 3 4 5 | 1. 2 3 4 5 |
| **5** | Provide lifestyle advice for modifiable risk factors | n.a. | 1 2 3 4 5 |

**The End**

Appendix 2b


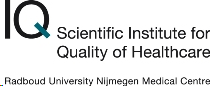

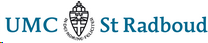


**Questionnaire for health professionals**


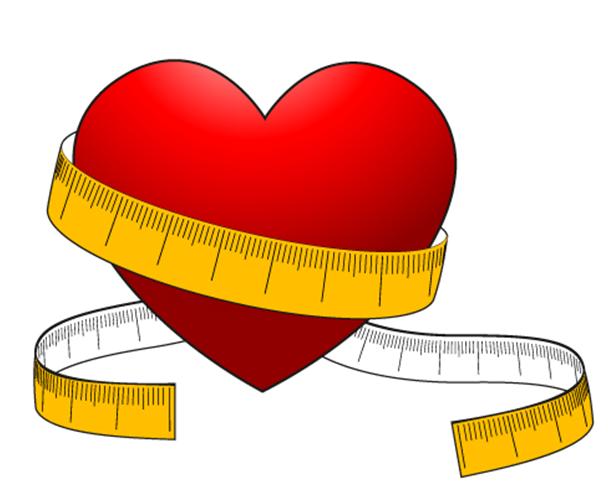


**Information networks**

**1.** We are interested in professional contacts between health care providers who are involved in cardiovascular risk management (CVRM). Please indicate in the scheme below to whom you have **given information** or from whom you have **receive information** in the past 12 months regarding:

1. Medical policy for CVRM in general, including special groups, such as patients of 80 years and older or with co-morbidity.
2. CVRM for a specific patient, e.g. when treatment is ineffective

- We also would like to ask you to indicate how often you’ve had contact for sharing information.

Please fill in a 1 for ‘daily or weekly’ and 2 for ‘monthly or yearly’

- We’ve already filled out names for persons from your general practice. Would you please fill out names for other persons you have shared information with?

|  | | **1.**  **Medical policy CVRM** | | How often contact for sharing information? | **2.**  **CVRM**  **For a specific patient** | | How often contact for sharing information? |
| --- | --- | --- | --- | --- | --- | --- | --- |
| **I gave information or received information from:** | | I gave to: | I received from: | 1= daily or weekly  2=monthly or yearly | I gave to: | I received from: | 1= daily or weekly  2=monthly or yearly |
| **Persons within a general practice:** | |  |  |  |  |  |  |
| GP1 | *Names* |  |  |  |  |  |  |
| GP2 |  |  |  |  |  |  |  |
| GP3 |  |  |  |  |  |  |  |
| PN1 |  |  |  |  |  |  |  |
| PN2 |  |  |  |  |  |  |  |
| PN3 |  |  |  |  |  |  |  |
| PA1 |  |  |  |  |  |  |  |
| PA2 |  |  |  |  |  |  |  |
| PA3 |  |  |  |  |  |  |  |
| **Persons outside a general practice:** | |  |  |  |  |  |  |
| Other GPs | *Names* |  |  |  |  |  |  |
| Other PNs |  |  |  |  |  |  |  |
| Other PAs |  |  |  |  |  |  |  |
| Dietician |  |  |  |  |  |  |  |
| Physical therapist |  |  |  |  |  |  |  |
| Psychologist |  |  |  |  |  |  |  |
| Cardiologist |  |  |  |  |  |  |  |
| Others, please specify: |  |  |  |  |  |  |  |
|  |  |  |  |  |  |  |  |

**1b.** In order to optimize knowledge on information flows of CVRM, we would to ask you share information with some questions as well. Therefore, we ask you to provide contact details (address, telephone number, or an e-mail address) of these persons. We emphasize that this information will be treated confidentially.

If you prefer not to provide contact details, please skip this question.

Contact details:

|  |  |  |
| --- | --- | --- |
|  |  |  |
|  |  |  |

**2**. Is someone responsible for coordination of CVRM in your organization? If yes, who is this person?

|  |  |  |
| --- | --- | --- |
|  |  |  |
|  |  |  |

**2b**. We also would like to ask some questions to this person(s). Therefore, we ask you to provide contact details (address, telephone number, or an e-mail address) of these persons. We emphasize that this information will be treated confidentially.

If you prefer not to provide contact details, please skip this question.

Contact details:

|  |  |  |
| --- | --- | --- |
|  |  |  |
|  |  |  |

**3.** Can you name one person (not an organization) who you consider to have a significant influence on your current practice in CVRM? This person can be anyone from inside or outside your organization, and the influence this person has had can be either current or from the past.

Please also provide a name an function.

| Name: |  |  |
| --- | --- | --- |
| Function: |  |  |
|  |  |  |

**3b**. We also would like to ask some questions to this person. Therefore, we ask you to provide contact details (address, telephone number, or an e-mail address) of this person. We emphasize that this information will be treated confidentially.

If you prefer not to provide contact details, please skip this question.

Contact details:

|  |  |  |
| --- | --- | --- |
|  |  |  |
|  |  |  |

**4.** We are interested in your opinion regarding recommendations for treatment of older patients (80 years and older) who receive care according to the CVRM guideline, and who have a limited life expectancy (less than 5 years).

Would you please indicate how important you consider:

- A change towards the treatment target, e.g. some reduction in blood pressure even though the treatment target has not been achieved
- Achieving the treatment target

Encircle a number: 1 = very unimportant, 2 = unimportant, 3 = neutral, 4 = important, 5 = very important.

|  | **Recommendation** | **Importance of change towards treatment target**  **(1-5)** | **Importance of achieving treatment target**  **(1-5)** |
| --- | --- | --- | --- |
| **1** | Aim at SBP < 140 mm Hg in patients with a 10 years risk of cardiovascular mortality or morbidity ≥ 20% | 1 2 3 4 5 | 1. 2 3 4 5 |
| **2** | Aim at SBP < 140 mm Hg for patients with CVD | 1 2 3 4 5 | 1. 2 3 4 5 |
| **3** | Aim at LDL cholesterol < 2.5 mmol/l in patients with a 10 years risk of cardiovascular mortality or morbidity ≥ 20% | 1 2 3 4 5 | 1. 2 3 4 5 |
| **4** | Aim at LDL cholesterol < 2.5 mmol/l for patients with CVD | 1 2 3 4 5 | 1. 2 3 4 5 |
| **5** | Provide lifestyle advice for modifiable risk factors | n.a. | 1 2 3 4 5 |

**The End**
