# Supplementary figures and images for: Social networks of health care providers and patients in cardiovascular risk management: a study protocol
Source: BMC Health Serv Res. 2014 Jun 18;14:265. doi: 10.1186/1472-6963-14-265 (PMC4071149; doi:10.1186/1472-6963-14-265)

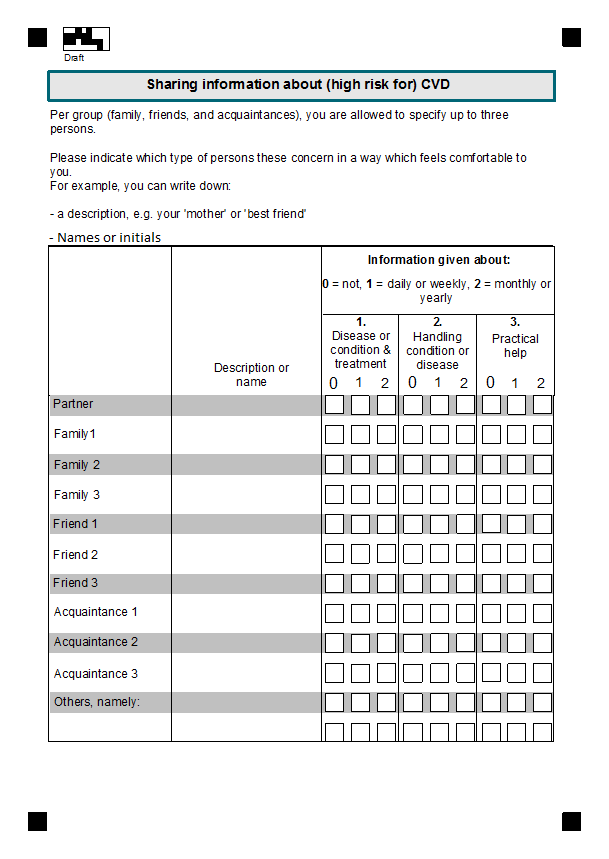

Supplement: Additional file 3 — Appendix 3a Network questionnaire for patients. Appendix 3b Network roster for alters of patients. [file 1472-6963-14-265-S3.zip › 1799078737113990_add5.docx]
